# Simultaneous Determination of 17 Pesticide Residues in Rice by GC/MS using a Direct Sample Introduction Procedure and Spiked Calibration Curves

**Published:** 2013

**Authors:** Maryam Amirahmadi, Hassan Yazdanpanah, Shahram Shoeibi, Morteza Pirali-Hamedani, Mahsa Ostad Gholami, Mohammad Farshid Mohseninia, Farzad Kobarfard

**Affiliations:** a *Department of Pharmacology and Toxicology, School of Pharmacy, Shahid Beheshti University of Medical Sciences, Tehran, Iran.*; b *Food and Drug Laboratories Research Center, Ministry of Health and Medical Education, Tehran, Iran. *; c *Food and Drug Control Laboratories, Food and Drug Deputy, Ministry of Health and Medical Education, Tehran, Iran. *; d *Department of Pharmacology, School of Pharmacy, Tehran University of Medical Sciences, Tehran, Iran.*; e *Department of Medicinal Chemistry, School of Pharmacy, Shahid Beheshti University of Medical Sciences, Tehran, Iran.*; f *Phytochemistry Research Center, Shahid Beheshti University of Medical Sciences, Tehran, Iran. *

**Keywords:** Pesticides, Spiked calibration curve, GC/MS, Rice

## Abstract

A reliable, rapid and accurate method based on spiked calibration curves and direct sample introduction was developed for determination of 17 pesticide residues in rice by gas chromatography-mass spectrometry single quadrupole selected ion monitoring GC/MS-SQ-SIM.

Sample preparation is based on extraction with acetonitrile without clean up. The use of spiked calibration curves for constructing the calibration curve substantially reduced adverse matrix-related effects.

The average recovery of pesticides at 6 concentration levels was in range of 97.5-102.1%. The method was proved to be repeatable with RSDr in range of 0.7%-19.8%for all of the concentration levels. The limits of detection and limit of quantifications for all the pesticides were < 10 ng/g and < 25 ng/g, respectively. The developed method was applied for simultaneous determination of the selected pesticides in 23 rice samples collected from Tehran retail market in March 2009.

Although many studies have been conducted regarding the determination of pesticides by using GC-MS, this is the first attempt in Iran using GC-MS-SIM technique that successfully can determine 17 pesticides with difference in physicochemical properties in rice.

## Introduction

The increasing public concern about pesticide contamination of food and the environment in recent years has increased the demand for broader and stricter pesticide monitoring (1). 

A few gas chromatography methods with specific detectors including electron capture detection (ECD) (2-5), flame photometric detector (FPD) (6), nitrogen phosphorus detection (NPD) (7), and mass spectrometer detectors (8-12), have been widely used for determination of pesticide residues in crops. Analysis of pesticide residues in rice has been the focus of a few recently published articles (8- 11). Different sample preparation processes have been employed in these articles including solid phase extraction (SPE), dispersive SEP (PSA) and gel permeation chromatography (GPC).

Recently, Anastassiades *et al. *proposed a simple, safe, cheap, high sample throughput method namely QuEChERS in pesticides residue analysis (13-18).

Generally, the complex matrix of agricultural products adversely affects analysis precision, and it is necessary to remove the matrix interference by sample pre-treatment, such as extraction and clean-up steps (19, 20). Since an effective elimination of the sources of the matrix-induced response enhancement is not likely in practice, the analysts often try to compensate for the effect using alternative calibration methods such as matrix mach calibration and standard addition methods (18).

More than 90% of the world’s rice is cultured and consumed in Asia (21). The consumption of rice in Iran is 110 g per capita/day (22).

This paper presents a rapid multiresidue method based on a direct sample introduction procedure (13) using spike calibration curve to simultaneously determine and confirm 17 pesticides in rice by gas chromatography with mass spectrometric detection by selected ion monitoring (GC/MS-SIM). The selected pesticides, included those for which MRL is issued by Institute of Standards of Iran (23), included iprodione, diazinon, carbaryl, fenthion, fenitrothion, pirimiphos-methyl, edifenphos, propiconazole, fipronil, chlorpyrifos, chlorpyrifos-methyl and oxadiazon. Propargite, dichlorvos, pirimicarb, malathion and fenvalerate were also added to the list of our pesticide of interest since they were some of found pesticides in rice by FDA during 1996-2006 (24).

The list of selected pesticides along with some of their physicochemical properties and their MRLs are presented in Table 1.

**Table 1:** Physicochemical properties and MRLs of the selected pesticides

**No. Compound**	**Structural group**	**M.F.**	**M.W.**	**MRL (ppm)**
1 Dichlorvos	Organophosphorus	C_4_H_7_Cl_2_O_4_P	220.98	-----
2 Diazinon	Organophosphorus	C_12_H_21_N_2_O_3_PS	304.35	0.20
3 Carbaryl	Carbamate	C_12_H_11_NO_2_	201.22	1.00
4 Fenitrothion	Organophosphorus	C_9_H_12_NO_5_PS	277.2	1.00
5 Fipronil	Phenylprazole	C_12_H_4_Cl_2_F_6_N_4_OS	437.2	0.01
6 Malathion	Organophosphorus	C_10_H_19_O_6_PS_2_	330.36	-----
7 Oxadiazon	Oxadiazole	C_15_H_18_Cl_2_N_2_O_3_	345.22	0.02
8 Primicarb	Carbamate	C_11_H_18_N_4_O_2_	238.00	------
9 *Chlorpyrifos	Organophosphorus	C_9_H_11_Cl_3_NO_3_PS	350.59	0.50
10 *Chlorpyrifos-methyl	Organophosphorus	C_7_H_7_Cl_3_NO_3_PS	322.5	0.10
11 Fenthion	Organophosphorus	C_10_H_15_O_3_PS_2_	278.33	0.05
12 Propiconazole	Triazole	C_15_H_17_Cl_2_N_3_O_2_	342.00	0.05
13 Propargite	Sulfite ester	C_19_H_26_O_4_S	350.00	-----
14 Edifenphos	Organophosphorus	C_14_H_15_O_2_PS_2_	310.4	0.10
15 Fenvalerate	Pyrethroid	C_25_H_22_ClNO_3_	419.92	-----
16 Pirimiphos-methyl	Organophosphorus	C_11_H_20_N_3_O_3_PS	305.00	1.00
17 Iprodione	Dicarboximide	C_13_H_13_CL_2_N_3_O_3_	330.17	^** ^10.00

In order to overcome the adverse matrix-related effects, it was decided to make the calibration standards by spiking blank rice samples with certain amounts of pesticides and constructing the calibration curve using these spiked calibration standards.

For application to real samples, twenty-three rice samples from local markets of Tehran in March 2009, were purchased and analyzed according to the validated method.

## Experimental

*Materials and Methods*

*Chemicals*

All pesticide standards were purchased from Dr. Ehrenstorfer Co. (Augsburg, Germany). All organic solvents, intended for extraction, were at least of LC grade and purchased from Merck (Darmstadt, Germany). Bulk quantities of anhydrous Na_2_SO_4_ and NaCl were obtained from Merck (Darmstadt, Germany).

*GC–SQ/MS*

An Agilent Technologies 6890N Network GC System chromatographs (Wilmington, USA) with a SQ detector and equipped with an Agilent 7683B autosampler (Agilent technologies, USA) was used. A HP-5 capillary column (30 m × 0.25 mm I.D., 0.25 μm film thicknesses) was used for separation.

*Calibration standards*

Individual stock standard solutions (1 mg/mL) were prepared in ethyl acetate and stored in dark at - 20°C. They were kept for 1 h at ambient temperature prior to their use. A mixed stock standard solution of pesticides was prepared in ethyl acetate at concentrations specified in Table 2.

**Table 2:** Concentrations of pesticides in mixed stock standard solution

**Concentrations (μg/mL)**						
0.6	1.2	3	6	12	30	60
Fipronil	Oxadiazon	Fenthion Propiconazole	Dichlorvos Primicarb Malathion Edifenphos Fenvalerate Propargite Chlorpyrifos-methyl	Diazinon	Chlorpyrifos	Carbaryl Iprodione Fenitrothion Pirimiphos-methyl

Spiked calibration standards at half maximum residue levels of 0.5 MRLs, 1 MRLs, 1.5 MRLs, 2 MRLs, 2.5 MRLs and 5 MRLs were prepared by addition of 250 μL, 500 μL, 750 μL, 1000 μL, 1500 μL and 2500 μL of mixed standard stock solution respectively to 30 g of blank rice samples in each case. In those pesticides for which no MRL has been set, mixed standard stock solutions were prepared according to Table 2.

A stock solution of triphenylmethane (TPM) in ethyl acetate at concentration of 1 mg/mL was used as internal standard. An aliquot of 20 μL of TPM solution in ethyl acetate (1000 mg/L) was added to the spiked rice sample as internal standard. The samples so obtained were treated as described in sample preparation section.

*Sample preparation*

For sample preparation, an aliquot of 20 μL of internal standard solution (1000 mg/L) was added to 30 g of rice sample in a warring blender and after being left for 1.5 h at ambient temperature in dark, 120 mL acetonitrile was added. The mixture was blended at high speed for 1 min. Six grams of NaCl was added to the mixture and blending was continued for an additional 60 sec. The slurry was transferred to a proper centrifuge tube and the residue in blender was rinsed with 50 mL acetonitrile and added to the centrifuge tube. After centrifugation for 7 min at 3000 rpm in - 5°C, the supernatant was filtered through 15 g of Na_2_SO_4_ and the filtrate evaporated to dryness. The residue was reconstituted in 1 mL toluene and 1 μL of the solution was injected into gas chromatograph.

*Recovery studies*

For recovery determination, spiked rice samples at concentration levels of 0.5, 1, 1.5, 2, 2.5 and 5 MRLs were prepared in triplicates and then treated according to the procedure described previously. The recoveries were calculated using the spiked calibration curves.

*GC-SQ-MS analysis*

The GC-SQ-MS was employed with helium as the carrier gas at a constant flow of 1 mL/min. The oven temperature started at 75°C and remained at this temperature for 3 min, then increased to 180°C at 25°C/min ramp rate and finally, increased to 300°C at 5°C/min ramp, holding at 300°C for 10 min. Injection port was adjusted at 250°C and splitless mode was used.

**Figure 1:**
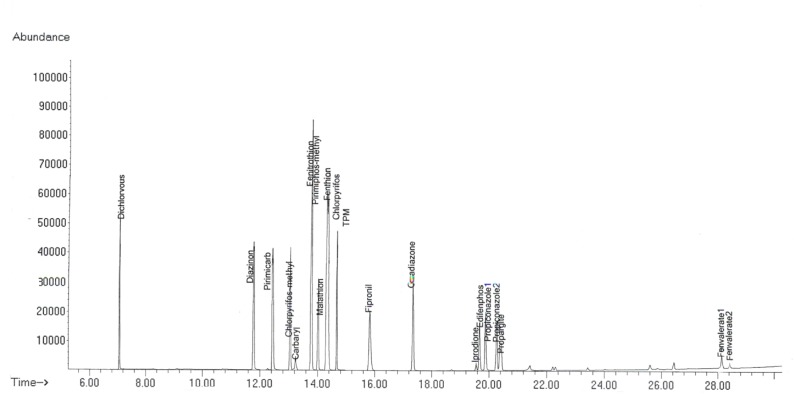
Representative chromatograms of 17 pesticides and internal standard.

After acquisition of the total ion chromatogram for the mixed stock standard solutions in scan mode, peaks were identified by their retention time and mass spectra. The identification was confirmed by comparing the relative abundances for three ions (one quantifier and two qualifiers) of the experimental standards to known relative abundances of the Pest Library reference spectra. The most abundant ion that showed no evidence of chromatographic interference and had the highest signal-to-noise ratio was taken for quantification purposes. A GC–SQ–MS chromatogram of 17 pesticides and internal standard (TPM) is shown in Figure 1.

*Quantitation*

The concentration of pesticides was determined by interpolation of the relative peak areas for each pesticide to internal standard peak area in the sample on the spiked calibration curve. In order to compensate for losses during sample processing and instrumental analysis, internal standard (TPM) was used.

*Application to real samples*

Twenty-three rice samples were purchased from local markets of Tehran in March 2009. In order to avoid any possible thermal decomposition of pesticide residues, 200 g rice sample was milled with Romer mill (Stylemaster Drive, USA) with 100 g dry ice. A 30 g portion of the powder was subjected to the process of sample preparation described previously.

## Results

*Gas chromatographic determination*

Analysis was performed in the SIM mode based on the use of one target and two qualifier ions. Pesticides were identified according to their retention times, target and qualifier ions. The quantitation was based on the peak area ratio of the targets to that of internal standard. Table 3 summarizes pesticides studied with their target and qualifier ions used in SIM mode in this study.

**Table 3:** The retention times, diagnostic ions and selected quantification ion for the target pesticides and internal standard.

**No.**	**Compound**	**Retention time**	**Diagnostic ions**	**Quantification ions **
1	Dichlorvos	7.66	185., 109, 220	109
2	Diazinon	11.87	304, 276, 179	304
3	Primicarb	12.45	238.2, 166.1	166.1
4	Chlorpyrifos-methyl	13.15	286, 125, 323	286
5	Carbaryl	13.29	144, 125.9, 115.1	144
6	Fenitrothion	13.87	277, 260, 214	277
7	Pirimiphos-methyl	13.88	305, 290, 276.1	305
8	Malathion	14.03	285, 173, 158	173
9	Fenthion	14.320	278, 169, 262.9	278
10	Chlorpyrifos	14.478	314, 197, 257.8	314
11	TPM (Istd)	14.699	244, 165	244, 165
12	Fipronil	15.89	367, 420, 351	367
13	Oxadiazon	17.548	344.1, 302, 258	258
14	Iprodione	19.57	244.1, 187, 161	187
15	Edifenphos	19.784	310, 200.9, 173	310
16	Propiconazole	19.89, 20.28	259, 190.9, 172.9	259
17	Propargite	20.433	350.2, 335.2, 201.1	350.2
18	Fenvalerate	28.13, 28.42	419.2, 225.1, 167	225.1

*Method validation*

*Linearity of the calibration curves*

Calibration curves were constructed for each compound using six different concentration levels. TPM was used as internal standard. For identification of pesticides, the retention time and three ions (one for quantitation and two for identification) were used.

The 17 pesticides showed linearity in SIM mode. Linear spiked calibration curves for all the pesticides were obtained with correlation factors more than 0.997. Table 4 shows calibration data (equation and regression coefficient) of interest pesticides in spiked rice calibration curves. 

**Table 4:** Calibration data (equation and regression coefficient) of 17 pesticides in spiked rice calibration curves.

**No.**	**Compound**	**Equation**	**Regression Coefficient **
1	Dichlorvos	y = 0.3026x - 0.0012	0.9996
2	Diazinon	y = 0.1518x - 0.0014	0.9996
3	Primicarb	y = 0.3101x + 0.0027	0.9985
4	Chlorpyrifos-methyl	y = 0.2629x - 0.0018	0.9993
5	Carbaryl	y = 0.2038x + 0.0029	0.9990
6	Fenitrothion	y = 0.0486x - 0.0094	0.9989
7	Pirimiphos-methyl	y = 0.0713x - 0.0138	0.9988
8	Malathion	y = 0.1023x + 0.0002	0.9973
9	Fenthion	y = 0.1256x + 0.0023	0.9988
10	Chlorpyrifos	y = 0.0385x - 0.0005	0.9998
11	Fipronil	y = 0.1361x - 2E-05	0.9982
12	Oxadiazon	y = 0.1407x - 0.0001	0.9978
13	Iprodione	y = 0.0458x + 0.0175	0.9995
14	Edifenphos	y = 0.1244x - 0.0021	0.9988
15	Propiconazole	y = 0.1634x + 0.0005	0.9988
16	Propargite	y = 0.0899x + 0.0004	0.9998
17	Fenvalerate	y = 0.0501x - 0.0005	0.9971

**Figure 2:**
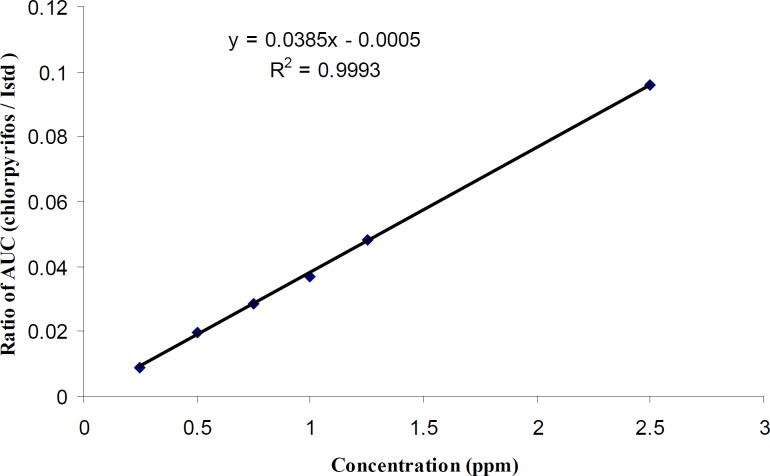
Spiked calibration curve for chlorpyrifos in rice sample

The spiked calibration curve of chlorpyrifos in rice sample is shown in Figure 2 as a representative.

*Limits of detection and limits of quantification*

Limits of quantification (LOQs) of the proposed method were calculated by considering a value 10 times that of background noise in spiked rice samples. The LOQs for all the pesticides in this method were calculated < 25 ppb.

*Recovery*

Table 5 presents the recovery and repeatability of 6 concentration levels. The recovery of pesticides at 6 concentration levels was in range of 97.48102.15%-. The method was proved to be repeatable with RSDr in range of 0.7%-19.8% at all spiking levels. The recoveries and repeatabilities are in accordance to the criteria set by SANCO Guideline (25).

**Table 5:** Average recoveries (%) and range of relative standard deviations (%) obtained by GC-MS analysis of rice at 6 spiking levels (n=3) in rice samples.

**Compound **	**Average recovery (%) (n=3) **							**Total Recovery ** **(%) (n=18) **	**Range of ** **RSD**_r_ **(%) **
	**0.5MRL **	**1MRL **	**1.5MRL**	**2MRL**	**2.5MRL**		**5MRL **		
Dichlorvos	95.6	108.5	99.5	104.3		103.3	99.5	101.79	2.4 - 16.7
Diazinon	102.4	101.3	94.9	100.0		102.4	99.7	100.16	3.9 - 10.8
Primicarb	87.2	99.7	100.0	97.5		107.1	98.7	98.40	1.7 - 19.3
Chlorpyrifos-methyl	103.6	98.8	95.9	98.6		104.1	99.5	100.12	3.4 - 13.7
Carbaryl	85.7	97.3	102.6	99.8		104.8	98.8	98.19	4.6 - 10.7
Fenitrothion	117.8	104.4	93.4	97.4		98.8	100.9	102.13	2.6 - 7.5
Pirimiphos-methyl	116.3	101.2	94.3	95.0		102.9	100.4	101.69	0.8 - 9.1
Malathion	104.5	105.4	85.8	101.7		105.1	99.4	100.35	2.5 - 19.1
Fenthion	80.2	104.8	106.7	101.7		96.7	99.9	98.36	3.3 - 15.9
Chlorpyrifos	103.0	103.9	98.2	98.4		99.9	100.3	100.62	2.1 - 9.3
Iprodione	88.9	113.2	100.6	99.7		99.2	99.8	100.25	2.7 - 10.7
Fipronil	91.7	109.5	99.1	92.7		104.6	99.8	99.58	4.9 - 16.1
Oxadiazon	121.8	100.0	92.7	93.2		104.7	100.3	102.15	3.5 - 15.5
Edifenphos	107.5	105.4	95.3	94.2		103.3	100.2	100.00	3.1 - 14.9
Propiconazole	117.8	94.8	93.7	98.4		103.7	99.9	101.40	3.6 - 11.8
Propargite	77.9	96.7	107.6	102.1		101.6	98.9	97.48	1.4 - 19.8
Fenvalerate	110.6	104.7	105.9	89.8		95.7	101.9	101.45	0.7 - 5.5

*Real samples*

**Figure 3:**
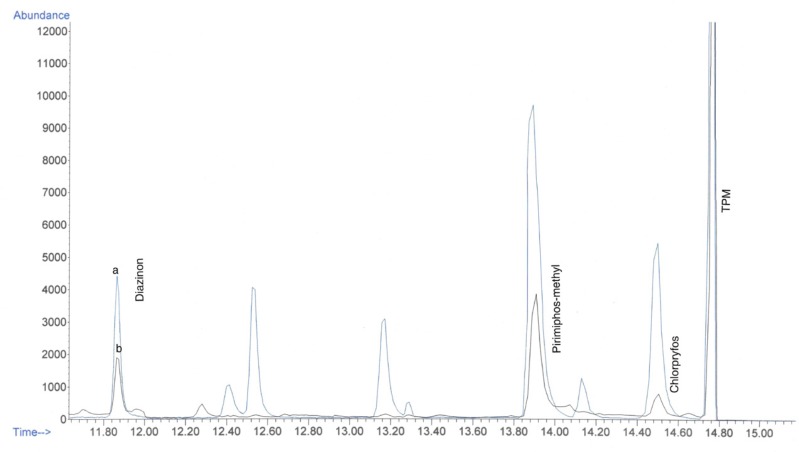
Chromatogram of (a) spiked rice sample at 1 MRL and (b) contaminated rice sample

For application to real samples, twenty-three rice samples were purchased from local markets of Tehran in March 2009 and analyzed according to the method described above. For evaluation of analysis, one QC sample at 1 MRL level was carried out in each working round. One of the 23 samples showed contamination with diazinon, pirimiphos-methyl and chlorpyrifos at concentrations of 0.10, 0.02, 0.09 ppm, respectively, which were below the MRLs of these pesticides in Iran. Figure 3 shows the overlaid chromatogram of a spiked rice sample at 1 MRL levels (a) and contaminated rice sample (b) in SIM mode.

## Discussion

Matrix-induced response enhancement is seemingly the most discussed matrix effect negatively impacting quantitation accuracy of certain analytes in GC (12). When a real sample is injected, the blocked of active sites (especially free silanol groups) in the GC inlet and column by the matrix components reduces losses of susceptible analytes caused by adsorption or degradation on these active sites. This phenomenon results in larger analyte signals in matrix-containing solutions in comparison to the matrix containing free solutions, which makes the convenient use of calibration standards in solvent only impractical. This would lead to overestimations of the calculated concentrations in the analyzed samples (12).

Another potential problem associated with matrix injections involves gradual accumulation of nonvolatile matrix components in the GC system, resulting in formation of new active sites and gradual decrease in analyte responses. This effect, sometimes called matrix-induced diminishment (26) negatively impacts ruggedness (*i.e.*, long-term repeatability of analyte peak intensities, shapes, and retention times), which is a highly important factor in routine GC analysis (17).

Theoretically, elimination of matrix components or active sites would surmount the matrix-induced enhancement effect; but, complete and permanent GC system deactivation or comprehensive sample cleanup is practically impossible (26, 27, 28).

Since an efficient elimination of the sources of the matrix is not possible in practice, the analysts are required to compensate for the effect using alternative calibration methods. The current compensation approaches include the use of the followings: (A) matrix-matched standards, (B) standard addition method, (C) isotopically labeled internal standards (not feasible in multiresidue pesticide analysis due to their unavailability or high price) and (D) usage of analyte protectants (18).

In the present study, we used spiked calibration standard approach to overcome the problems caused by the matrix. In this approach, calibration standards are prepared by the addition of standard solution to blank rice samples that are subjected to the same sample preparation procedure which is intended to be used for unknown samples. In this way, the standard sample matrices will have the same composition as the unknown samples and therefore the effect of matrix is reflected in both standards and unknown samples. The calibration curve is constructed using these spiked calibration standards and it is easily used to calculate the concentration of analyte(s) in unknown sample without being concerned about the matrix effects. 

## Conclusion

A simple and rapid method was developed to determine 17 pesticide residues in rice; a main food in Iranian food basket. 

The proposed method not only allowed the simultaneous determination and confirmation of 17 pesticides with good recoveries and low detection limits, but also showed to be useful in routine analysis due to its fast and easy procedure. 

The developed method has the advantage of using spiked calibration curves that minimize the matrix interferences leading to higher accuracy for pesticides analyses. 
